# Commentary: Microbial Resistance Movements: An Overview of Global Public Health Threats Posed by Antimicrobial Resistance, and How Best to Counter

**DOI:** 10.3389/fpubh.2020.629120

**Published:** 2021-01-20

**Authors:** Mohammed S. Razzaque

**Affiliations:** Department of Pathology, Lake Erie College of Osteopathic Medicine, Erie, PA, United States

**Keywords:** microbial resistance, antibiotics, Public Health, antimicrobial stewardship, global healh

## Introduction

“Superbugs” are the antimicrobial-resistant microorganisms. Bacteria, viruses, fungi, and parasites acquire the ability to evade the antimicrobial drug effects, not only to survive but also, in some cases, become more virulent. As such, the existing antimicrobial drugs are no longer effective and useful in treating the infections (used to be treatable). Superbug-induced infections are the major worldwide health concern with higher human mortality and an increased financial burden on society. The underlying mechanism of the evolvement of drug-sensitive to drug-resistant microorganisms is an extremely complex phenomenon. It is partly related to microorganism's unique ability to modify their genetic structures and biochemical functionality to survive and keep growing even in the presence of antimicrobial drugs. Since there are multiple factors involved in developing antimicrobial drug resistance, it cannot be reversed by adopting a single prevention strategy. Of importance, certain bacterium may not require antimicrobial drug exposure to develop resistance, as surrounding environmental exposure can facilitate the resistance.

Consistent misuse and overuse of antimicrobial drugs by healthcare professionals and consumers with its extensive use in food and meat production have put human health at risk. Lack of resources for research and low interest in developing the newer generation of antimicrobial drugs are also contributing to the evolution of superbugs. Without global involvement, partnership and collaboration, superbug-induced morbidity and mortality will be unmanageable in the future. To address this impending global health crisis, in May 2015, the World Health Organization (WHO) assembly adopted a global action plan to combat the antimicrobial resistance, that include (1) to increase the awareness of antimicrobial resistance, (2) to advance research and surveillance, (3) to cut down the rate of infections through preventive measures, (4) to ensure the optimal use of antimicrobial drugs, and (5) to develop sustainable investment, taking into account the needs of the countries, to develop novel interventions. Unfortunately, implementing such WHO measures have both financial and logistic hurdles, and the rates of superbug-induced infections are alarmingly increasing.

**Figure 1:**
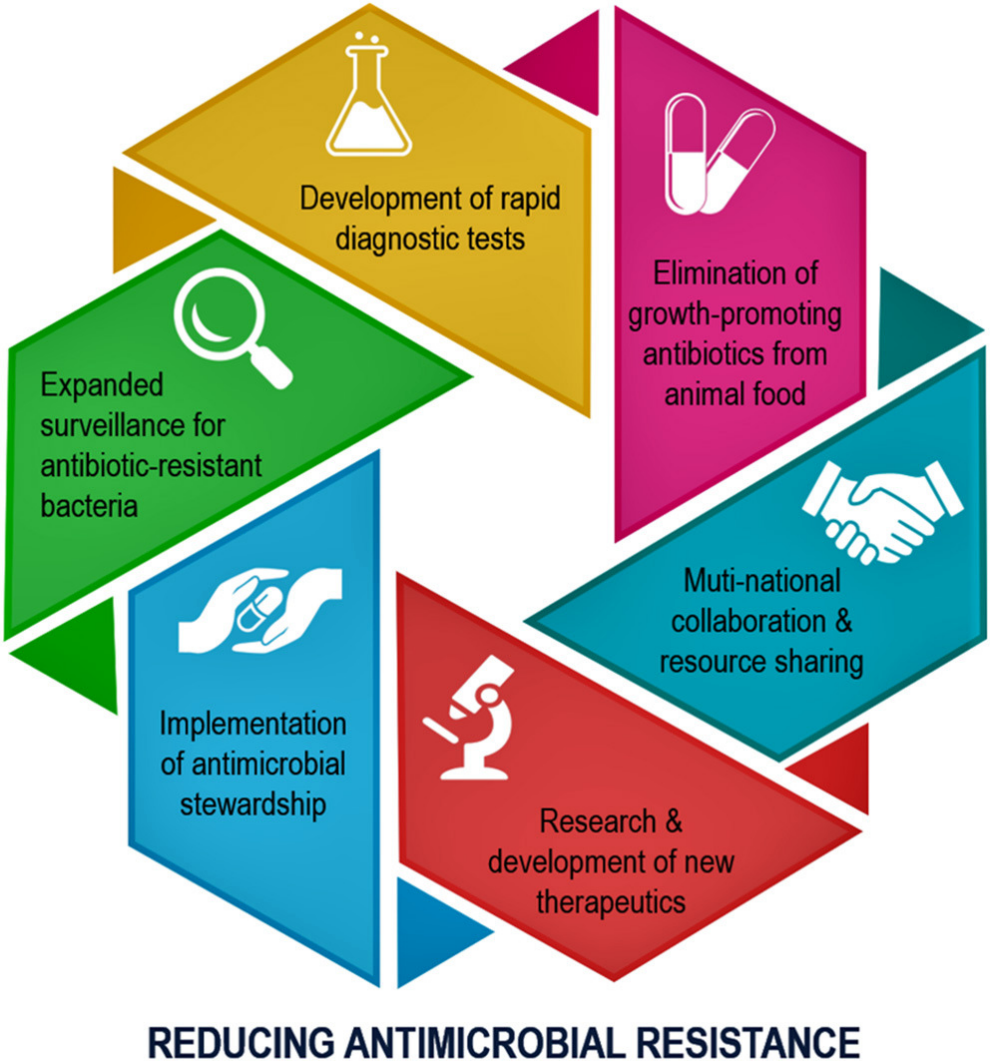
Schematic diagram showing the main steps needed to be implemented to minimize the antimicrobial drug resistance (2).

A recently published article in “Frontiers in Public Health” has highlighted the importance of microbial resistance movements to reduce the burden of superbug-induced infections (1). The authors have elaborated on the genesis of antimicrobial drugs and listed the challenges of producing the newer generation of drugs to combat existing drug-resistant pathogens (1). Although the authors briefly touched on the irrational prescription of antimicrobial drugs in the both developed and developing countries, the review article did not discuss in depth the regulatory enforcement procedures to minimize the antimicrobial resistance (1). The opportunities and challenges of global standardization of the antimicrobial prescription processes were not explained in detail in the publication (1). Another critical area that was not emphasized enough in the article was the use of the antimicrobial drugs on animals and its potential consequences in developing antimicrobial resistance. This commentary will briefly elaborate on the cross-species transmission of resistant pathogens from animal to human. Appreciating that one single review article cannot cover all the aspects of antimicrobial resistance, the authors have fittingly highlighted the scientific and economic challenges that are hindering the novel antimicrobial drug development (1). The publication has justifiably concluded that antimicrobial resistance is a multifaced issue driven by numerous interrelated factors, and therefore, the use of any single intervention would have limited success (1). Recent publications have also emphasized why implementing an antimicrobial stewardship program is necessary to prepare the future medical professionals to enhance their awareness and knowledge of antimicrobial resistance to reduce the disease burden related to superbug-mediated infections (Figure 1) (2, 3).

### Antimicrobial Stewardship

Antimicrobial stewardship program is one of the most effective approaches to educate healthcare professionals to select suitable antimicrobial medications for required patients for the right period to lower the emergence of antimicrobial resistance (4). Of concern, antimicrobial drugs are commonly used in clinical practice, and around 50% of antibiotics prescribed in the hospitals are unnecessary (5). In a similar line of observation, the Centers for Disease Control and Prevention (CDC) reported that during 2010–2011, around 154 million times, antibiotics were prescribed in the ambulatory care settings in the U.S., of which ~47 million were estimated as unnecessary or inappropriate prescriptions (6). Such an irrational use of antimicrobial drugs partly contributed to the development of resistance against the microorganisms, once treatable before the emergence of resistance (7, 8). One unfortunate example would be the treatment of gonorrhea. Azithromycin and ceftriaxone were very effective in treating gonorrhea. However, gonorrhea is no longer responsive to azithromycin and ceftriaxone treatment due to their overuse/ misuse and the subsequent development of antimicrobial resistance. The CDC categorized gonorrhea as an “urgent threat” (9, 10). Therefore, it is of utmost importance to implement antimicrobial stewardship programs to educate future healthcare professionals on the responsible use of antimicrobial drugs to improve their effectiveness and sustainability (11). As mentioned, in the U. S., the annual (2010–2011) antibiotic prescription was 506 per 1,000 population, but only 353 antibiotic prescriptions were estimated to be appropriate (6). Implementing an antimicrobial stewardship program makes it possible to reduce around 30% fewer antibiotic prescriptions yearly, which will have far-reaching impacts and benefits on human health. Although the unnecessary antimicrobial prescriptions in humans with subsequent drug exposure are among the major causes of antimicrobial resistance for specific strains, (needless) prescriptions alone are not the cause of antimicrobial resistance for all bacterial strains (12).

### Antimicrobial Drug Use on Livestock

Another important area that requires intense focus to reduce antimicrobial resistance is the use of antibiotics to promote animal growth for meat consumption. In the U.S., around 70–80% of clinically important and useful antibiotics are sold and utilized for the maintenance and growth of the livestock (13). Such massive use of antibiotics on meat-producing animals is likely to promote antimicrobial resistance, although animal to the human association is not universally accepted and contested by certain groups. However, studies have shown that when foodborne pathogens (Campylobacter) were challenged with antimicrobial drugs, resistant pathogens could be isolated from the exposed animals (14). Of more concern, antimicrobial resistance remained higher for years, even after discontinuation of antibiotics (14). How quickly microorganisms can gain resistance usually depends on the microbial strains and the drugs used (15). Even though the resistance of animal pathogens on farms can risk human health is an ongoing area of research; a U.S. study found that tetracycline-treated birds (broiler chicken) resulted in the emergence of tetracycline resistance pathogens, which were traced in the birds, and isolated from 11 members of the farms who were exposed to those housed birds (16). The study suggests the chain of events of how antimicrobial resistance can develop in the birds, and can eventually be transmitted to humans. The cross-species transmission of resistant pathogens from animal to human can be transmitted by direct contact between humans and animals by consuming contaminated food or sharing pathogen-polluted water (17). According to the CDC, more than 35,000 yearly deaths are estimated to be related to antibiotic-resistant infections in the U.S., costing more than US$50 billion (18). It is essential to realize that even if unnecessary antimicrobial prescriptions in humans are reduced, it may not necessarily eliminate the antimicrobial resistance altogether, as the use of antimicrobial drugs on animals can also initiate and propagate antimicrobial resistance. From the available evidence, it appears to be rational to advocate reducing or more preferably eliminating growth-promoting antimicrobial use in animals and birds to minimize the occurrence of antimicrobial resistance (19). Of relevance, such initiatives are partially implemented in Europe and should be executed globally. Decreasing antibiotic use reduces the prevalence of antimicrobial resistance in animals by around 15% and multidrug-resistant bacteria by ~24–32% (19). The subsequent effect on humans is difficult to determine, but it is estimated that around a 24% reduction in the prevalence of antimicrobial resistance in humans with reduced use of antimicrobial drugs in animals (19).

## Conclusion

Naturally occurring antimicrobial resistance is a prolonged and slow process. On the other hand, the overuse or misuse of antimicrobial drugs can rapidly induce antimicrobial resistance, and these superbugs are clinically challenging to eradicate. Through the antimicrobial stewardship program, necessary training should be provided to the healthcare professionals to reduce the unnecessary use of antimicrobial drugs to delay the occurrence of antimicrobial-resistant organisms. Also, alternatives to the existing antimicrobial drugs or supplementary agents are needed to reduce the burden of antimicrobial drug resistance. For instance, a recent publication from McGill University (in Montreal, Canada) has claimed that a cranberry extract can make the bacteria more sensitive to antibiotic treatment (20). Developing clinically viable quorum sensing inhibitors to supplement existing antimicrobial agents would be another research avenue to pursue (21, 22). Finally, the empiric antibacterial therapy during the ongoing COVID-19 pandemic is likely to enhance antibiotic-resistant microorganisms (19–21). There is an urgent need to develop more potent antibiotics and/or innovative therapeutic strategies to deal with such emerging microbial resistance (23–25). The newly developed therapeutics need to be strictly regulated to avoid the past error of overuse-antimicrobial resistance. The coordinated and collaborative efforts among the national and international governmental and private agencies are required to achieve substantial progress in reducing or delaying the occurrence of antimicrobial resistance.

## Author Contributions

MR: conceptualized and wrote the article.

## Conflict of Interest

The author declares that the research was conducted in the absence of any commercial or financial relationships that could be construed as a potential conflict of interest.
